# Global Burden and Trends of Norovirus-Associated Diseases From 1990 to 2019: An Observational Trend Study

**DOI:** 10.3389/fpubh.2022.905172

**Published:** 2022-06-17

**Authors:** Xiaobao Zhang, Can Chen, Yuxia Du, Danying Yan, Daixi Jiang, Xiaoxiao Liu, Mengya Yang, Cheng Ding, Lei Lan, Robert Hecht, Shigui Yang

**Affiliations:** ^1^State Key Laboratory for Diagnosis and Treatment of Infectious Diseases, National Clinical Research Center for Infectious Diseases, Collaborative Innovation Center for Diagnosis and Treatment of Infectious Diseases, The First Affiliated Hospital, Zhejiang University School of Medicine, Hangzhou, China; ^2^Department of Big Data Health Science, School of Public Health, Zhejiang University, Hangzhou, China; ^3^Department of Epidemiology of Microbial Diseases, Yale School of Public Health, New Haven, CT, United States

**Keywords:** norovirus-associated diseases, Global Burden of Disease, epidemic features, death rate, global trend analysis

## Abstract

**Introduction:**

As an important pathogen causing diarrheal diseases, the burden and change in the death rate of norovirus-associated diseases (NADs) globally are still unknown.

**Methods:**

Based on global disease burden data from 1990 to 2019, we analyzed the age-standardized death rate (ASDR) of NADs by age, region, country, and Socio-Demographic Index (SDI) level. The discrete Poisson model was applied in the analysis of NADs' spatiotemporal aggregation, the Joinpoint regression model to analyze the trend of death burden of NADs over 30 years, and a generalized linear model to identify the risk factors for the death rate from NADs.

**Results:**

The ASDR of NADs significantly decreased by a factor of approximately 2.7 times, from 5.02 (95% CI: 1.1, 11.34) in 1990 to 1.86 (95% CI: 0.36, 4.16) in 2019 [average annual percent change (AAPC) = −3.43, 95% CI: −3.56, −3.29]. The death burden of NADs in 2019 was still highest in African regions despite a great decline in recent decades. However, the ASDR in high SDI countries presented an uptrend [0.12 (95% CI: 0.03, 0.26) in 1990 and 0.24 (95% CI: 0.03, 0.53) in 2019, AAPC = 2.52, 95% CI: 2.02–3.03], mainly observed in the elderly over 70 years old. Compared to children under 5 years old, the 2019 death rate of elderly individuals over 80 years old was much higher in high SDI countries. The generalized linear model showed that factors of the number of physicians (RR = 0.67), the proportions of children under 14 years old (RR = 1.21), elderly individuals over 65 years old (RR = 1.13), educational level (RR = 1.03) and urbanization proportion (RR = 1.01) influenced the ASDR of NADs.

**Conclusions:**

The death burden of NADs has remained high in developing regions over the last three decades and has increased among the elderly in countries with high SDI levels, even though the global trend in NAD-associated deaths has decreased significantly in the past three decades. More effective public health policies against NADs need to be implemented in high SDI regions and for the elderly.

## Introduction

Although the disability-adjusted life years (DALYs) and age-standardized death rate (ASDR) of diarrheal diseases decreased by 51% and 49%, respectively, from 1990 to 2010, diarrheal diseases remain the fourth cause of DALYs and seventh cause of death, resulting in approximately 1.45 million deaths worldwide every year (1, 2). Previous studies suggested that diarrheal diseases were the second leading cause of death among children under 5 (3–5). They represented 78% of all diarrhea deaths among children in the African, Southeast Asian, and Eastern Mediterranean regions (6). The mortality rates of diarrheal diseases in the developing regions referenced above were over 25% (4). According to an attribution study of diarrheal diseases, norovirus was a common pathogen of diarrheal diseases, with 13.8% of children under 5 years requiring hospitalization, second only to rotavirus (38.3%) and enteropathogenic Escherichia coli (15.3%) (7). Norovirus was considered related to almost one-fifth of all acute gastroenteritis cases across all age groups (8, 9). Patel MM et al. estimated that norovirus was detected in 12% of children under 5 years of age with severe diarrhea, which indicated that norovirus was the second most common cause of severe gastroenteritis after rotavirus (10). In developed countries that introduced a rotavirus vaccine program, norovirus surpassed rotavirus as the leading cause of acute gastroenteritis in children (11). The U.S. Centers for Disease Control and Prevention (CDC) also suggested that norovirus caused approximately 60% of acute gastroenteritis cases (with a known cause) in the United States every year (12).

However, insufficient attention has been given to the detection, diagnosis, and surveillance of norovirus. Payne et al. searched ICD-9-CM discharge diagnostic codes and found that there was no ICD-9-CM code for norovirus (008.63) among 278 laboratory-confirmed norovirus cases (13). Previous studies on the disease burden were limited in data by the effect of annual and seasonal epidemics and varied greatly in investigated age groups and locations (14). Consequently, high-quality studies on the disease burden, trends, and epidemic features of norovirus-associated diseases (NADs) are still missing, especially in developing regions. Based on global disease burden data, we investigated the global death burden and trend of NADs worldwide with the stratification of multiple factors from 1990 to 2019 and explored the correlations between ASDR and sociodemographic economic factors. This study aims to better understand the disease burden of NADs and identify the regions and populations with high death rates or those on the rise, providing insight to assist targeted policymaking and recommending actions to reduce the death rate of NADs.

## Materials and Methods

### Data Sources

Referring to the categories of diarrhea rates reported in clinical, case-control, and community studies, Global Burden of Disease (GBD) 2019 estimated the death numbers and rates of NADs globally by age, sex, cause, region, and Socio-Demographic Index (SDI) level (7). The Bayesian hierarchical meta-regression tool (Dismod-MR) and the Cause of Death Ensemble model (CODEm) were used to calculate the estimates of metrics. And the death rate of NADs was calculated from diarrhea data via a counterfactual approach called population attributable fraction (PAF) (15). We obtained the data from GBD 2019 using the Global Health Data Exchange, including the number of deaths, death rate, and ASDR of NADs with 95% uncertainty intervals (UIs) across 204 countries and territories and 21 GBD regions from 1990 to 2019. Jiang et al. obtained the genome of norovirus in 1993 (16). After that, molecular methods like the RT-PCR were developed. Therefore, the global screening data of NADs relying on molecular diagnostic techniques in the 1990s is likely to be biased. To eliminate the influence of data bias on the results, we made an additional analysis of data from 2005 to 2019. A total of 204 countries were divided into five SDI quintiles (high, high-middle, middle, low-middle, and low) according to economic growth, fertility rate, and educational attainment. The SDI ranges from 0 to 1, and the standard of classification was provided by GBD studies. All rates are reported per 100,000 person-years.

### Spatial and Temporal Aggregation Analysis

The geographical distribution and spatiotemporal aggregation were performed to explore the space-time cluster of NADs worldwide from 1 January 1990 to 31 December 2019. Based on the discrete Poisson model, national units and time were scanned via the dynamic space-time two-dimensional cylinder scanning window. For each scanning window, the expected number of deaths was estimated from the actual number of deaths and the population. The log-likelihood ratio (LLR) and relative risk (RR) calculated from the actual and expected death numbers were used for estimating the presence and degree of aggregation. The cluster was classified according to the LLR value. The window with the largest significant LLR value statistically was the first cluster. *P*-values were calculated by Monte Carlo simulation, with *P* < 0.05 considered significant.

### Joinpoint Regression Model Analysis

Joinpoint regression program (17) was developed by the National Cancer Institute (NCI). The Joinpoint regression model helped find the turning points that were statistically significant via the Monte Carlo permutation test. We used Joinpoint Regression Software (18) to calculate the annual percent change (APC) with 95% CIs and the average annual percent change (AAPC) to analyze the temporal trends of the age-standardized death rate of NADs by SDI quintiles. An APC or AAPC greater or smaller than 0 denoted an up or downtrend of the ASDR. The level of significance was set at *P* < 0.05.

### Generalized Linear Model

Based on previous studies, we selected 5 covariates that were statistically significant from 14 country-level sociodemographic covariates across 199 countries from 1990 to 2019. We used several types of generalized linear models to fit the age-standardized death rate and each country-level sociodemographic covariate. The best-fit model with a Gaussian-distributed corresponding variable was determined according to the Akaike information criterion. Fitted model equation:


$$\begin{array}{l} \begin{array}{l} Log[E(Yt)]=\beta 0+\beta 1(<14\text{-}year\;proportion)\\ \;+\beta 2(>65\text{-}year\;proportion)+\beta 3(Urban\;proportion)\\ \;+\beta 4(Physicians)+\beta 5(Educational\;level) \end{array} \end{array}$$

where Yt denotes the age-standardized death rate; β0 is the intercept; β (<14-year proportion), β (>65-year proportion), β (urban proportion), β (physicians), and β (educational level) denote the population proportion under 14 and over 65 years old, the proportion of urbanization, the number of physicians per 1,000 persons and the average level of education, at least bachelor's or equivalent, respectively.

### Software

We used Microsoft Excel 2019 for data extraction, sorting, and cleaning and R (version 3.2.3), SatScan (version 9.5), and Joinpoint (version 4.8.0.1) for further data analysis.

## Results

### The Number and ASDR of NADs in 1990 and 2019 With Change Rate and AAPC Over the 30 Years

In total, there were 135,798 NADs deaths worldwide in 2019, and the ASDR was 1.86 (95% UI: 0.36–4.16) per 100,000 person-years. Children younger than 5 years old (0.65, 95% UI: 0.18–1.49) and elderly individuals at least 80 years old (0.7, 95% UI: 0.08–1.76) had a higher ASDR than others (Table 1). The ASDR declined in most of the 21 GBD regions, whereas Australasia, high-income North America, and Western Europe increased from 1990 to 2019 with significant AAPCs (Table 1, Supplementary Figure S1).

**Table 1:** The number and age-standardized death rate (ASDR) of norovirus-associated diseases (NADs) in 1990 and 2019 with change rate and average annual percent changes (AAPCs) over the 30 years.

	**1990**		**2019**			
**(l0ptr0pt)2-3 (l0ptr0pt)4-5**	**Death number**	**Age-standardized death**	**Death number**	**Age-standardized death**	**Change in**	**AAPC, % (95%CI)**
	**(95%UI)**	**rate (95%UI)**	**(95%UI)**	**rate (95%UI)**	**rate, %**
Global	242996 (57060–548907)	5.02 (1.10–11.34)	135798 (303735–25103)	1.86 (0.36–4.16)	−62.95	−3.43* (−3.56,−3.29)
**Gender**	
Male	124373 (30915–276475)	5.57 (1.19–12.78)	67057 (13337–153741)	1.97 (0.39–4.55)	−64.63	−3.51* (−3.57,−3.46)
Female	118623 (26092–272770)	4.69 (0.95–10.80)	68741 (12142–164092)	1.76 (0.32–4.21)	−62.47	−3.32* (−3.47,−3.17)
**Age group**	
<5	136696 (39387–303549)	21.63 (6.23–48.02)	43481 (11754–99172)	6.56 (1.77–14.96)	−69.67	−4.04* (−4.19,−3.89)
5–14	9717 (1085–25417)	0.87 (0.10–2.27)	5849 (624–15090)	0.45 (0.05–1.16)	−47.93	−2.22* (−2.50,−1.93)
15–49	16627 (1696–41140)	0.61 (0.06–1.52)	12544 (1251–31495)	0.32 (0.03–0.80)	−48.00	−2.21* (−2.44−1.97)
50–69	27192 (2975–67475)	3.99 (0.44–9.89)	19170 (2056–46517)	1.39 (0.15–3.37)	−65.13	−3.56* (−3.79−3.32)
≥70	52764 (6032–126429)	26.18 (2.99–62.73)	54755 (5914–136950)	11.81 (1.28–29.54)	−54.89	−2.71* (−2.95−2.46)
**SDI rank**	
High SDI	1098 (207–2341)	0.12 (0.03–0.26)	5494 (657–12080)	0.24 (0.03–0.53)	100.00	2.52* (2.02, 3.03)
High-middle SDI	5783 (1483–12209)	0.59 (0.15–1.26)	2791 (394–6328)	0.17 (0.03–0.37)	−71.19	−4.20* (−4.43,−3.98)
Middle-SDI	53049 (14029–107909)	4.46 (0.99–9.40)	22808 (4103–50232)	1.17 (0.22–2.59)	−73.77	−4.54* (−4.61,−4.46)
Low-middle SDI	98573 (22421–225670)	13.67 (2.32–31.61)	46984 (8259–112305)	3.87 (0.59–9.67)	−71.69	−4.27* (−4.55,−3.99)
Low SDI	84352 (17902–195657)	21.76 (3.59–50.93)	57647 (11446–131692)	8.04 (1.26–18.84)	−63.05	−3.38* (−3.50,−3.25)
**GBD regions**	
Andean Latin America	2052 (525–4093)	5.25 (1.20–11.02)	473 (85–1087)	0.84 (0.15–1.95)	−84.00	−6.33* (−6.62,−6.05)
Australasia	8 (1–18)	0.04 (0.01–0.09)	51 (6–118)	0.09 (0.01–0.21)	125.00	2.89* (1.35, 4.44)
Caribbean	1840 (524–3898)	5.08 (1.44–10.70)	765 (165–1644)	1.75 (0.39–3.75)	−65.55	−3.61* (−4.15,−3.07)
Central Asia	1564 (478–3380)	1.71 (0.52–3.71)	163 (39–360)	0.18 (0.05–0.40)	−89.47	−7.41* (−7.82,−6.99)
Central Europe	76 (23–158)	0.08 (0.02–0.17)	157 (20–367)	0.08 (0.01–0.17)	0.00	−0.25 (−2.10, 1.64)
Central Latin America	10778 (3112–20052)	7.52 (1.87–14.47)	2759 (513–5685)	1.23 (0.23–2.52)	−83.64	−6.11* (−6.24,−5.97)
Central Sub-Saharan Africa	9808 (2138–24517)	21.53 (3.77–51.27)	6775 (1371–15837)	8.46 (1.36–19.97)	−60.71	−3.11* (−3.31,−2.90)
East Asia	9704 (2718–20124)	0.92 (0.25–1.97)	665 (127–1567)	0.05 (0.01–0.12)	−94.57	−9.73* (−10.11,−9.35)
Eastern Europe	271 (74–554)	0.14 (0.04–0.29)	94 (17–198)	0.04 (0.01–0.08)	−71.43	−4.17* (−5.59,−2.73)
Eastern Sub-Saharan Africa	32348 (7381–75613)	21.89 (3.78–52.95)	18334 (3637–40247)	7.64 (1.13–17.64)	−65.10	−3.56* (−3.79,−3.33)
High-income Asia Pacific	457 (68–865)	0.31 (0.05–0.59)	1489 (195–2943)	0.25 (0.03–0.48)	−19.35	−0.75* (−1.26,−0.24)
High-income North America	118 (21–255)	0.03 (0.01–0.07)	2215 (264–5039)	0.32 (0.04–0.72)	966.67	8.36* (7.59, 9.12)
North Africa and Middle East	10090 (2765–23020)	2.3 (0.59–5.25)	2867 (766–6713)	0.56 (0.14–1.31)	−75.65	−4.76* (−4.95,−4.56)
Oceania	202 (39–477)	6.75 (0.94–17.15)	251 (42–622)	3.71 (0.48–9.57)	−45.04	−2.03* (−2.20,−1.86)
South Asia	80257 (15885–183923)	16.63 (2.33–39.31)	47125 (6836–119350)	4.31 (0.56–11.26)	−74.08	−4.54* (−4.97,−4.10)
Southeast Asia	27385 (6353–62262)	9.58 (1.64–22.27)	12280 (1802–28849)	2.58 (0.37–6.10)	−73.07	−4.43* (−4.52,−4.33)
Southern Latin America	231 (62–477)	0.52 (0.13–1.08)	230 (31–511)	0.28 (0.05–0.62)	−46.15	−2.07* (−2.56,−1.58)
Southern Sub-Saharan Africa	9132 (2607–17444)	22.51 (5.18–44.17)	6849 (1368–13898)	12.11 (2.14–26.04)	−46.20	−2.15* (−2.35,−1.95)
Tropical Latin America	3524 (1041–7586)	2.64 (0.75–5.62)	850 (170–1938)	0.41 (0.09–0.92)	−84.47	−6.25* (−6.48,−6.02)
Western Europe	340 (47–770)	0.06 (0.01–0.15)	2106 (246–4874)	0.18 (0.02–0.41)	200.00	4.05* (3.46, 4.65)
Western Sub-Saharan Africa	42809 (8932–102605)	21.93 (3.83–51.61)	29299 (5785–66497)	8.08 (1.41–18.54)	−63.16	−3.39* (−3.56,−3.23)

### Global Burden of NADs Among 204 Countries and Territories

**Figure 1:**
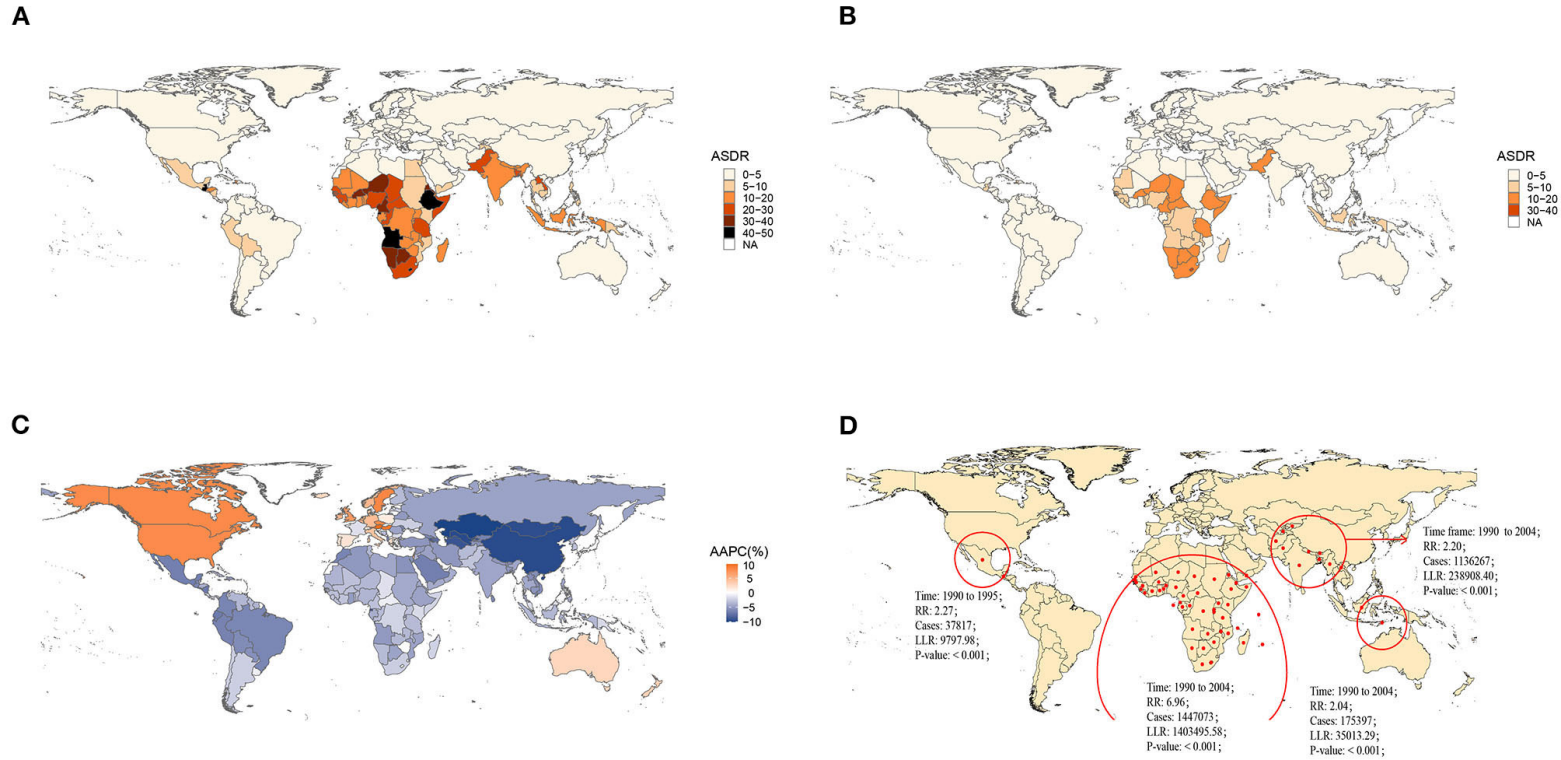
Global burden of norovirus-associated diseases (NADs) in 1990 and 2019 with the annual percent change rate and spatial and temporal aggregation over the 30 years. **(A)** Age-standardized death rate (ASDR) in 1990; **(B)** ASDR in 2019; **(C)** average annual percent changes (AAPCs) from 1990 to 2019; **(D)** Spatial and temporal aggregation from 1990 to 2019.

Nationally, Lesotho showed the highest ASDR in 1990, followed by Angola. Austria and Greece had the lowest ASDR in 1990 (Figure 1A, Supplementary Figures S2–S6). By 2019, Lesotho still presented the greatest age-standardized death rate, and Greece still had the lowest ASDR (Figure 1B, Supplementary Figures S2–S6). Regionally, African regions had higher ASDRs in 1990 and 2019 (Figures 1A,B). Globally, the ASDR of most countries and territories decreased significantly over the past 30 years, except for North America, Western Europe, and Australia (Figure 1C). The ASDR in East Asia (AAPC = −9.45%, *P* < 0.05) presented the largest drop and the greatest annual percent change at the regional level. In contrast, the ASDR in high-income North America (AAPC = 8.19%, *P* < 0.05) increased with the greatest AAPC (Supplementary Figure S1).

Four spatial and temporal aggregation areas were observed through spatial and temporal aggregation analyses. The first-level spatial and temporal aggregation areas were mainly located in African regions, and the gathering time was from 1 January 1990 to 31 December 2004. The actual number of cases reported in the regions was 1,447,073, while the number of expected cases was 268,296 (RR = 6.96, LLR = 1,403,495.58, *P* < 0.001). The second and third spatial and temporal aggregation areas were both in South Asia from 1 January 1990 to 31 December 2004, with relative risks of 2.2 (LLR = 238,908.40, *P* < 0.001) and 2.04 (LLR = 35,013.29, *P* < 0.001), respectively. The fourth spatial and temporal aggregation area covered Mexico and Guatemala from 1 January 1990 to 31 December 1995. The actual number of cases reported was 37,817, but the number of expected cases was 16,728 (RR = 2.27, LLR = 9,797.98, *P* < 0.001) (Figure 1D).

### Age-Specific Temporal Trends of NADs Burden Among SDI Quintiles From 1990 to 2019

**Figure 2:**
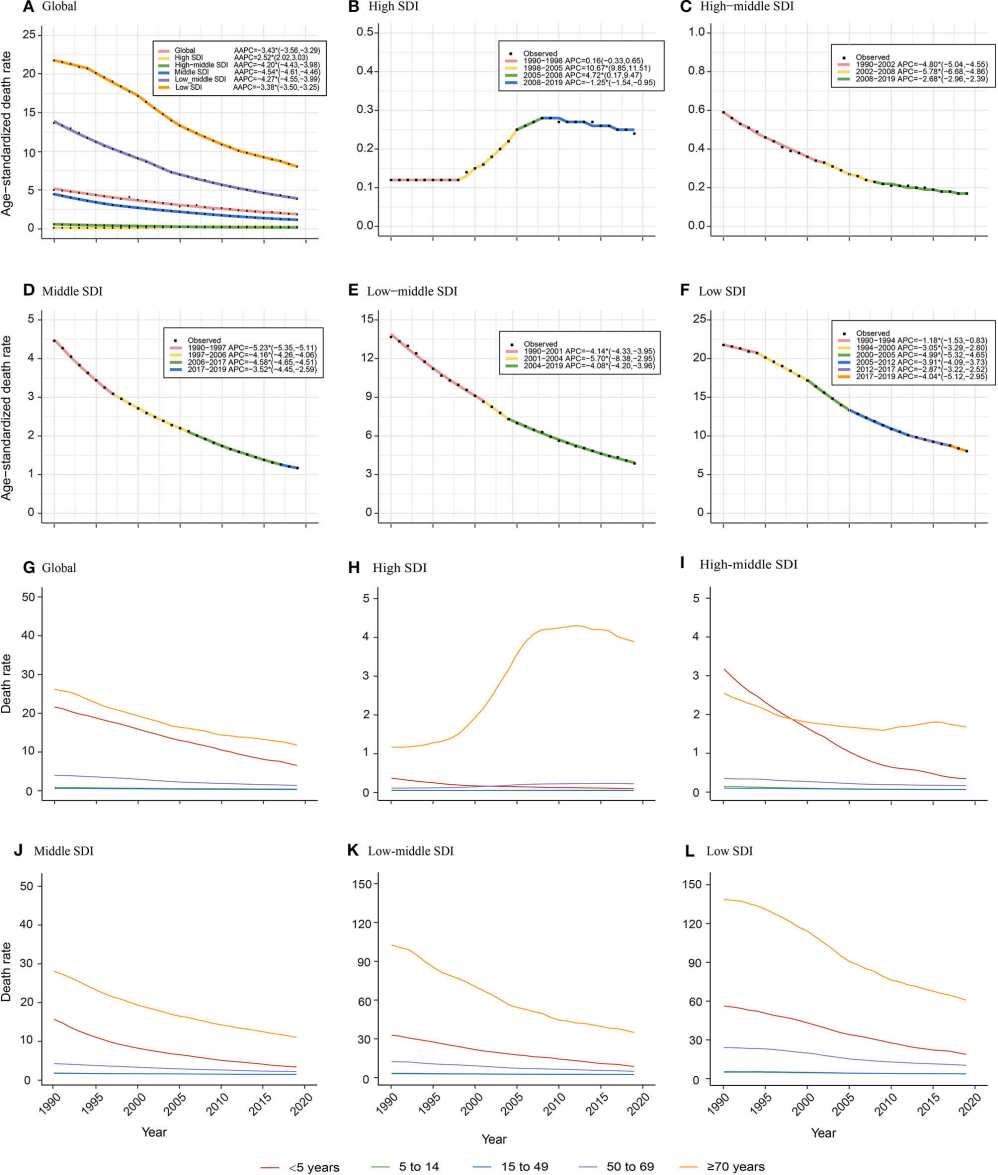
Age-specific temporal trends of ASDR globally and in different Socio-Demographic Index (SDI) regions over 30 years old. **(A–F)** Temporal trends of ASDR globally and in different SDI regions; **(G–L)** Age-specific temporal trends of death rate globally and in different SDI regions. The APCs with asterisks (*) are statistically significant (*P* < 0.05).

Socio-Demographic Index-stratified joinpoint analysis showed that the ASDR of NADs declined significantly since 1990 globally and in high-middle, middle, low-middle, and low SDI regions (Figure 2, Table 1). The proportions of NADs in diarrheal disease-associated deaths showed a decreasing or stabilizing trend in those regions (Supplementary Figures S7, S9–S12). However, the ASDR in high SDI regions presented different trends. As shown in Figure 2B, the ASDR showed an uptrend with different APCs. Between 1998 and 2005, the ASDR rose rapidly (APC = 10.67%, *P* < 0.05). Then, between 2005 and 2008, the ASDR continued to increase significantly (APC = 4.72%, *P* < 0.05). Since 2008, the rate presented a slight downtrend, however, it was still higher than that in the past, approximately doubling the burden compared to 1990 (Figure 2B). The proportion of NADs in diarrheal disease-associated deaths increased from 12.24% in 1990 to 15.69% in 2019, which has surpassed rotavirus to rank second among 13 pathogens in high SDI regions (Supplementary Figure S8).

Globally, children under 5 years old and the elderly over 70 years old have born with the heaviest death burden from NADs over the 30 years old. The death rate of children younger than 5 years old presented a significant decrease since 1990. The death rate of the elderly over 70 years decreased globally and in high-middle, middle, low-middle, and low SDI regions. However, it showed an increasing trend among elderly over 70 years old showed in high SDI regions (Figure 2G).

### Age Group Distribution of NADs Burden in 1990 and 2019 Among SDI Quintiles and Worldwide

**Figure 3:**
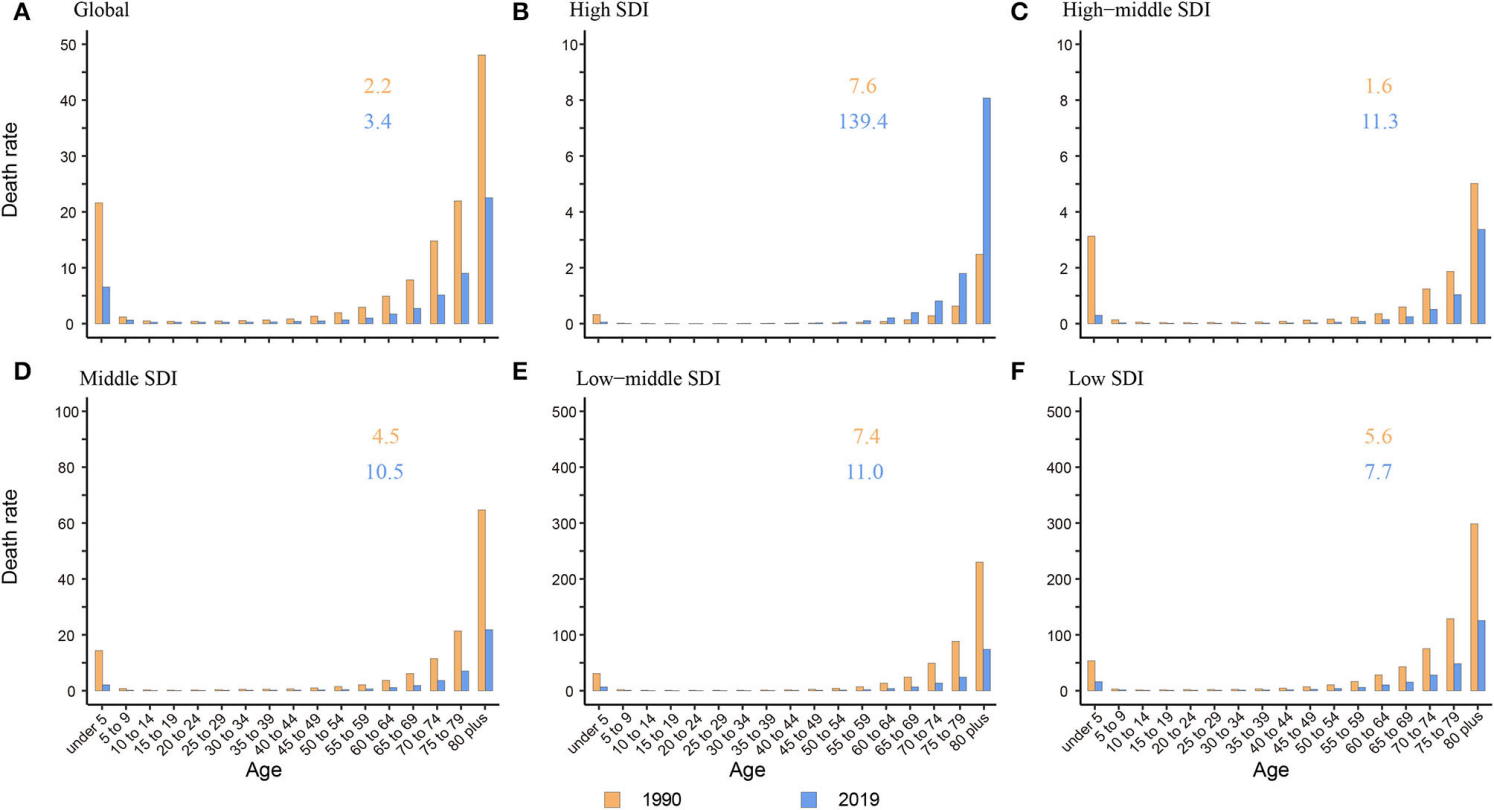
The death rate of NADs among children under 5 and elderly over 80 globally and in 5 SDI regions. **(A)** Global; **(B)** High SDI; **(C)** High-middle SDI; **(D)** Middle SDI; **(E)** Low-middle SDI; **(F)** Low SDI. The orange and blue numbers above the column denote the ratio of elderly over 80 years old to children under 5 years old in 1990 and 2019 separately.

The death rate was the highest in the elderly over 80 years old among all age groups globally and in five SDI regions both in 1990 and 2019. In adults and the elderly, the death rate presented a tendency to increase with age. Children under 5 years old were another peak population with a high death rate. The ratio of elderly over 80 years old to children under 5 years old increased from 1.6 to 7.6 in 1990 and 3.4 to 139.4 in 2019, which showed a growing trend in the proportion of death burden in elderly over 80 years old among all age groups (Figure 3). Compared to other SDI regions, the ratio was much higher in the high SDI region with a ratio of 139.4 (3.4 to 11.3 in other SDI regions) (Figure 3B).

### Correlation Analysis of Sociodemographic Factors With the ASDR of NADs

The generalized linear model (GAM) found that the number of physicians per 1,000 persons had a significant negative effect on the ASDR of NADs, RR = 0.67 (95% CI: 0.58, 0.78). In contrast, the proportion of individuals under 14 (RR = 1.21) and over 65 years old (RR = 1.13), urbanization proportion (RR = 1.01) and average national educational level (RR = 1.03) were significantly positively correlated with the ASDR (Table 2). Furthermore, in low SDI regions, the ASDR declined sharply with the increasing SDI index. The downward trend flattened in the middle and high SDI regions (Supplementary Figure S13A). A correlation analysis showed that there was a negative correlation (R = −0.63, *P* < 0.05) between the ASDR of NADs and the SDI index at the national level in 2019 (Supplementary Figure S13B).

**Table 2:** The correlation analysis of sociodemographic factors to the ASDR of NADs.

**Coefficients**	**Estimate**	**Std. error**	***P*–value**	**RR (95%CI)**
Intercept	−7.04	0.57	<0.05	-
<14-year proportion	0.19	0.01	<0.05	1.21 (1.18, 1.24)
>65-year proportion	0.12	0.02	<0.05	1.13 (1.10, 1.17)
Physicians	−0.40	0.07	<0.05	0.67 (0.58, 0.78)
Urban proportion	0.01	0.00	<0.05	1.01 (1.00, 1.02)
Educational level	0.03	0.01	<0.05	1.03 (1.01, 1.05)

## Discussion

Despite greatly declining over several decades, the ASDR in developing areas (e.g., Africa, and Southeast Asia) was still highest, adding up to more than 80% of the ASDR globally, which was consistent with that of previous studies (Supplementary Figure S1). Norovirus is mainly transmitted through the fecal-oral route with several other transmission routes, aerosolized viral particles (19, 20), food, water, and environmental pollution (21). The malnutrition and lack of vitamins, substandard quality of water, insufficient health facilities, and prolonged breastfeeding over 6 months in those areas could affect the incidence and death rate from diarrheal disease in different proportions (22–24). Compared to developed areas, diarrheal patients in developing areas may obtain fewer medical resources for pathogen detection and further treatment. Oral rehydration salts (ORS) and zinc supplementation are critical for diarrheal patients, whereas the coverage of drugs remains relatively low across the developing world, partly explaining the high death rate in these areas (25).

Due to the development of diagnostic techniques and medical interventions, the ASDR of diarrheal diseases in high-middle, middle, low-middle, and low SDI regions has fallen substantially in recent decades (Figures 2C-F). One unexpected finding was that the ASDR in high SDI regions, high-income North America, Western Europe, and Australasia presented a significant uptrend after 1998 (Figure 2B, Supplementary Figure S1). The ASDR of NADs surpassed rotavirus as the second-highest among 13 pathogens of infectious diarrhea in high SDI regions in 2019 (Supplementary Figure S8). Previous studies showed that the global prevalence of norovirus gastroenteritis has been associated with a single genotype named GII.4 since the 1990s (26–29). GII.4 strains caused several main outbreaks of norovirus between 1995 and 2008 with a 2- to the 3-year interval (30). The first wave was in 1995/1996 and was caused by the U5-95_US strain (26, 31–33). and then Australia in 1997–2000 (27). In 2002, the new GII.4 variants of “the Farmington Hills virus” caused epidemics in Europe (34), the United States (28), and Australia (26). In 2004, the “Hunter virus,” another GII.4 variant emerged and caused multiple outbreaks in Australia, New Zealand, Japan and Taiwan, and Europe (35, 36). According to Kroneman A, GII.4 genotype noroviruses were dominant in the United States, Europe, and Oceania from 1998 to 2007, accounting for 70%-80% of all outbreaks (37). Another study showed that hospitalizations and deaths were more likely to occur in outbreaks related to GII.4 strains, with an incidence ratio of 9.4 and a mortality rate of 3.1 (38). The outbreaks of the GII.4 variant in these developed countries might explain the increase of the death rate from 1990 to 2008 in high SDI regions (26, 39). In the rotavirus vaccine era, the burden rank of norovirus in 13 pathogens causing diarrheal diseases has gradually increased. Norovirus infection was shown to be more prevalent than rotavirus infection in children in the US recently (13). Similar increases have been observed in other countries (11, 40, 41). A longitudinal epidemiological study in the United States compared the positive rates of norovirus and rotavirus in the feces of diarrheal children before and after the introduction of the rotavirus vaccine in 8.5 years and concluded that the total prevalence of norovirus was 10.9% and increased after 2003, between 11% and 16.8% (42). More attention to norovirus is needed, including dedicating attention to the development of norovirus vaccines.

Like many other diseases, children and the elderly are the two age groups having relatively poor immunity and are more vulnerable to bacteria and viruses. We found that in high-middle, middle, low-middle, and low SDI regions, there were two death rate peaks in 2019: children under 5 and elderly individuals over 80 years old (Figure 3). Hall et al. also reported that norovirus-related hospitalization rates are u-shaped, and the incidence is highest among children under 5 years old (9.4 per 10,000) and over 65 years old (8.1 per 10,000) (11). Interestingly, in the high SDI region, there was only one death rate peak in 2019 of those over 80 years old without an insignificant young age peak. The death rate of children under 5 years old was almost equal to that of children over 5 years old and adults (Figure 3B). One plausible explanation is that the level of medical and health care in high SDI regions is relatively higher, lowering the death rate of children under 5 years. Additionally, with better medical and health security, more elderly individuals live in nursing homes and hospitals in high SDI areas. In an interesting coincidence, a previous study demonstrated that GII.4 was more likely to be transmitted via person-to-person transmission, especially in long-term care facilities (LTCFs) and hospital settings, and GI.7 and GII.12 were more likely to be transmitted via foodborne transmission. Moreover, in a total review of 2,895 outbreaks with known transmission routes in the United States from September 2009 to August 2013, person-to-person and foodborne transmission accounted for 83.7% and 16.1%, respectively (43). In the study by Scallan E and his colleagues, the proportion of foodborne noroviruses declined from 40% to 26%, consistent with estimates in other studies (44–46). The poor immunity, GII.4 strains, person-to-person transmission, and specific places all indicate that elderly individuals are currently the most vulnerable people. In addition, the growing death rate of the elderly in high SDI areas illustrates the need for more attention and interventions in older people in developed countries (Figure 2H).

As an important embodiment of medical resources, the assignment of doctors per 1,000 persons was closely associated with the death rate of diarrheal diseases. Previous studies have suggested that there is a negative correlation between the incidence of diarrheal diseases and educational level in developing countries (47–49). People with higher educational levels may have enough nutrition, health knowledge and good personal hygiene to prevent infections. There is less chance for these people to both transmit and be infected by norovirus. However, in the result of our generalized linear model, a higher ASDR was associated with a higher educational level (Table 2). This finding is supported by a population-based cohort study in the Netherlands (9). de Wit et al. explained that people who receive more education may also have higher incomes. They eat out more and have more opportunities to try specialty foods. Another study also showed that highly educated people were more likely to have risk behaviors of foodborne infection (50). In this study, the proportion of urbanization was positively correlated with the ASDR (Table 2). An explanation for this observation is that the population density in urban areas is higher. People residing in urban areas are more likely to have interpersonal transmission. A cross-sectional study of Haitian children also observed that children residing in urban areas reported diarrhea much more frequently than those from rural areas (51). High SDI regions and countries had lower ASDRs, also denoting that we need to make more effort on policy and medical interventions in low SDI areas (Supplementary Figure S13). The risk factors in the results can provide some suggestions for the specific implementation aspects.

### Limitation

The death rate perhaps says more about the severe cases among all norovirus-infected patients. Although being proportional to the incidence, the death rate only partly reflects the burden and epidemic features of NADs. It is better to understand the disease burden of NADs from multiple indicators. The modeled data of norovirus in the GBD study was estimated based on the vital registration, verbal autopsy data, and surveillance system data. However, the detection of norovirus depended on the development of molecule diagnostics like RT-PCR, so the data from surveillance systems in the 1990s and early 2000s was probably biased, especially in the areas with scarce medical sources.

## Conclusion

We analyzed the global death burden and epidemic features of NADs in different regions and countries among people of different ages. The overall burden of NADs is on the decline except for the high SDI region and the elderly. The death burden in developing areas is still much higher than that in developed areas. Overall, improving medical care in these places, strengthening the protection of vulnerable populations, and accelerating the development of targeted vaccines are important for easing the disease burden of NADs.

## Data Availability Statement

The original contributions presented in the study are included in the article/Supplementary Material, further inquiries can be directed to the corresponding author/s.

## Author Contributions

SY designed the study. XZ, CC, YD, DY, DJ, XL, MY, CD, and LL collected data. XZ and CC analyzed data. XZ and YD checked the data and results. SY, XZ, CC, and DY interpreted the data and wrote the report. RH modified the language. SY and RH revised the report from the preliminary draft to submission. All authors have read and agreed to the published version of the manuscript.

## Funding

This study was supported by grants from the National Natural Science Foundation of China (grant numbers: 82173577, 81672005, U1611264, and 81001271) and the Mega-Project of National Science and Technology for the 12th and 13th Five-Year Plan of China (grant numbers: 2018ZX10715-014-002 and 2014ZX10004008).

## Conflict of Interest

The authors declare that the research was conducted in the absence of any commercial or financial relationships that could be construed as a potential conflict of interest.

## Publisher's Note

All claims expressed in this article are solely those of the authors and do not necessarily represent those of their affiliated organizations, or those of the publisher, the editors and the reviewers. Any product that may be evaluated in this article, or claim that may be made by its manufacturer, is not guaranteed or endorsed by the publisher.
