# Supplementary figures and images for: Global Burden and Trends of Norovirus-Associated Diseases From 1990 to 2019: An Observational Trend Study
Source: Front Public Health. 2022 Jun 17;10:905172. doi: 10.3389/fpubh.2022.905172 (PMC9247406; doi:10.3389/fpubh.2022.905172)

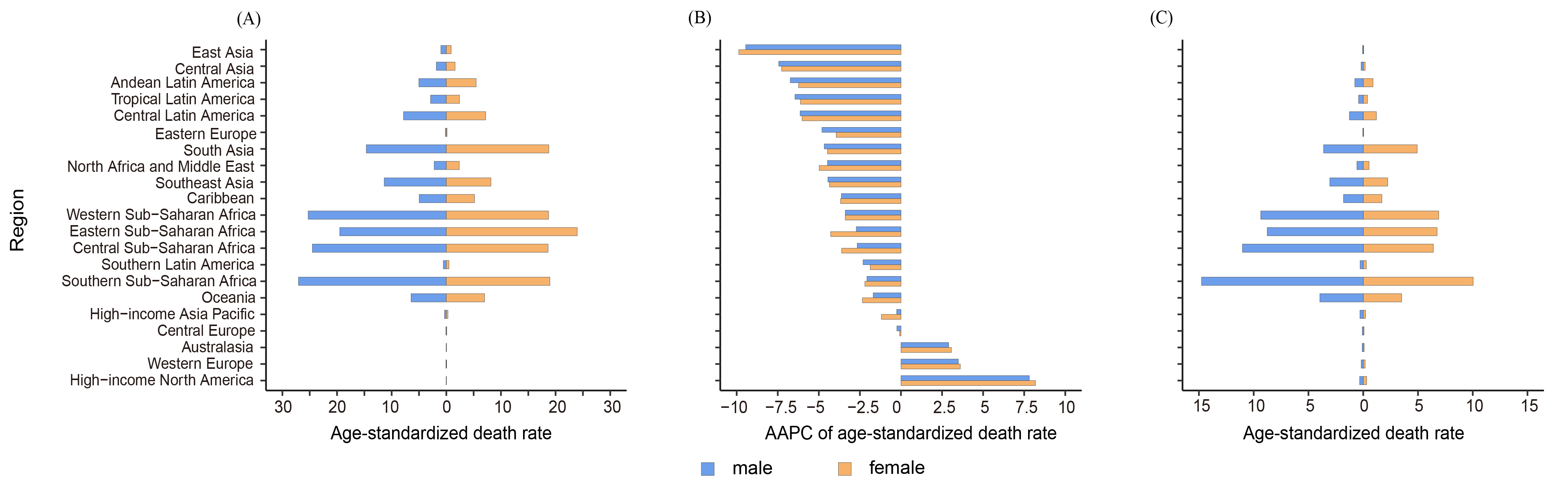

Supplement: Supplementary file 1 [file Data_Sheet_1.ZIP › Supplemental Materials/Supplementary Figure.S1.tif]

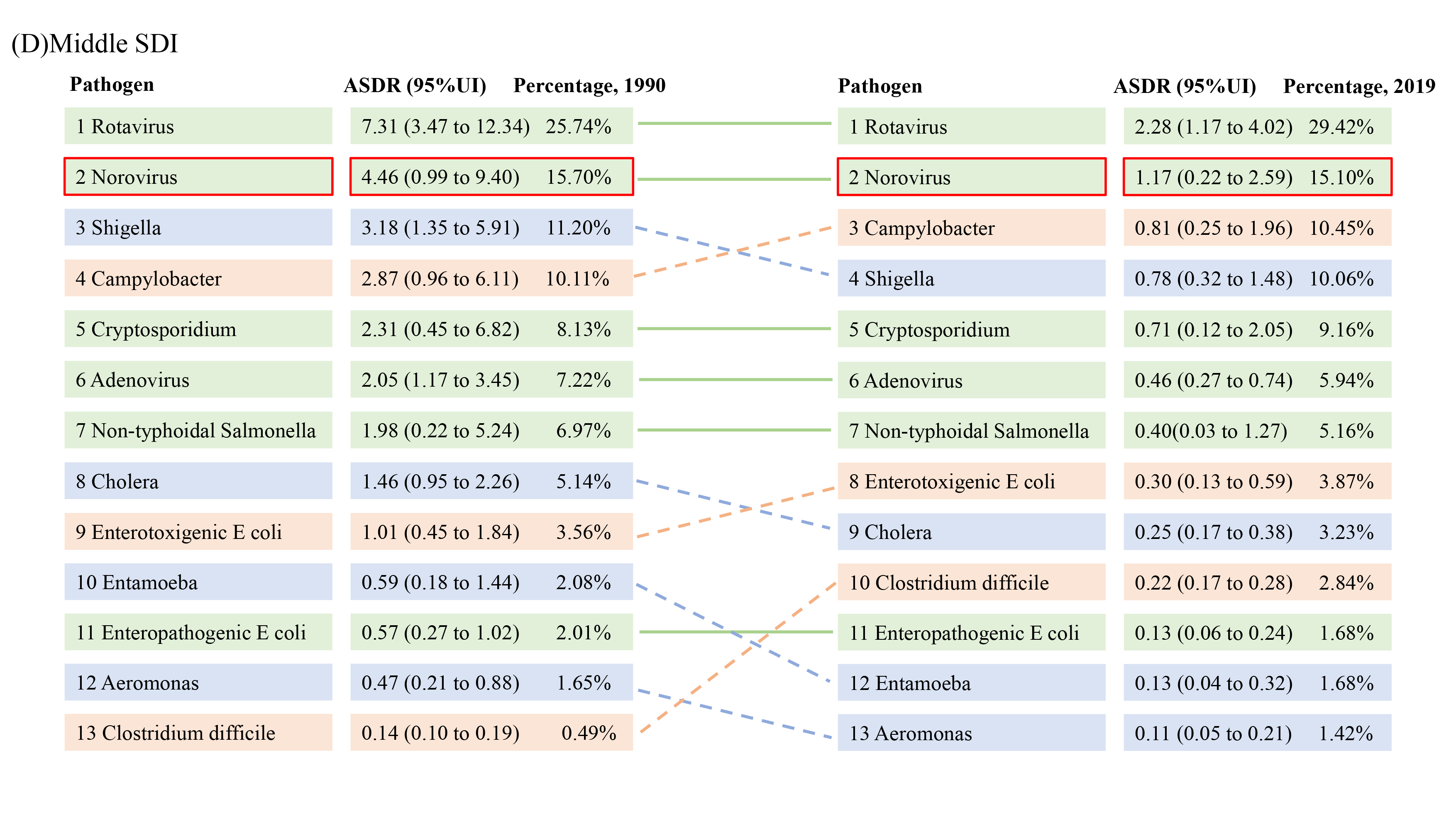

Supplement: Supplementary file 1 [file Data_Sheet_1.ZIP › Supplemental Materials/Supplementary Figure.S10.tif]

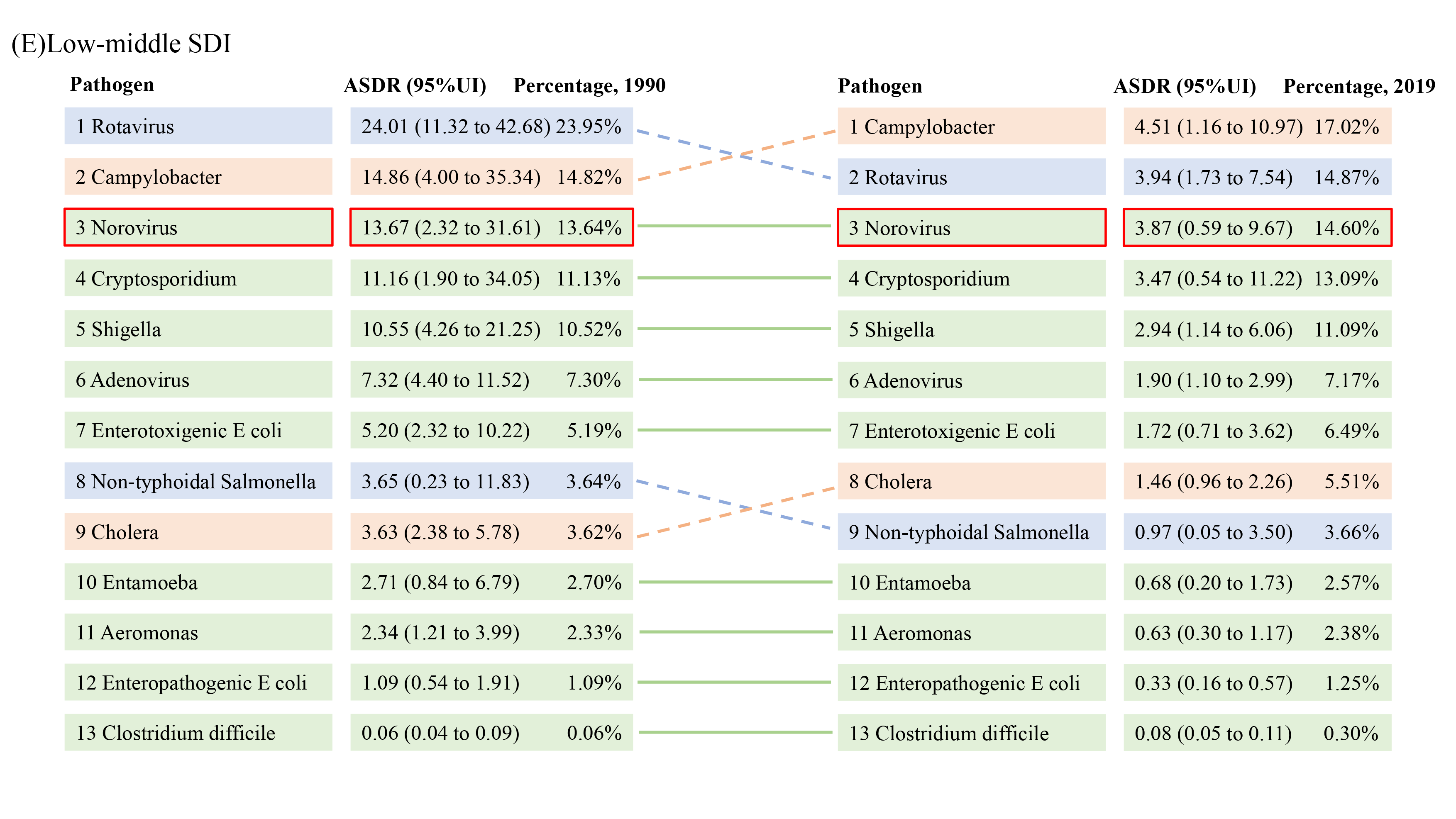

Supplement: Supplementary file 1 [file Data_Sheet_1.ZIP › Supplemental Materials/Supplementary Figure.S11.tif]

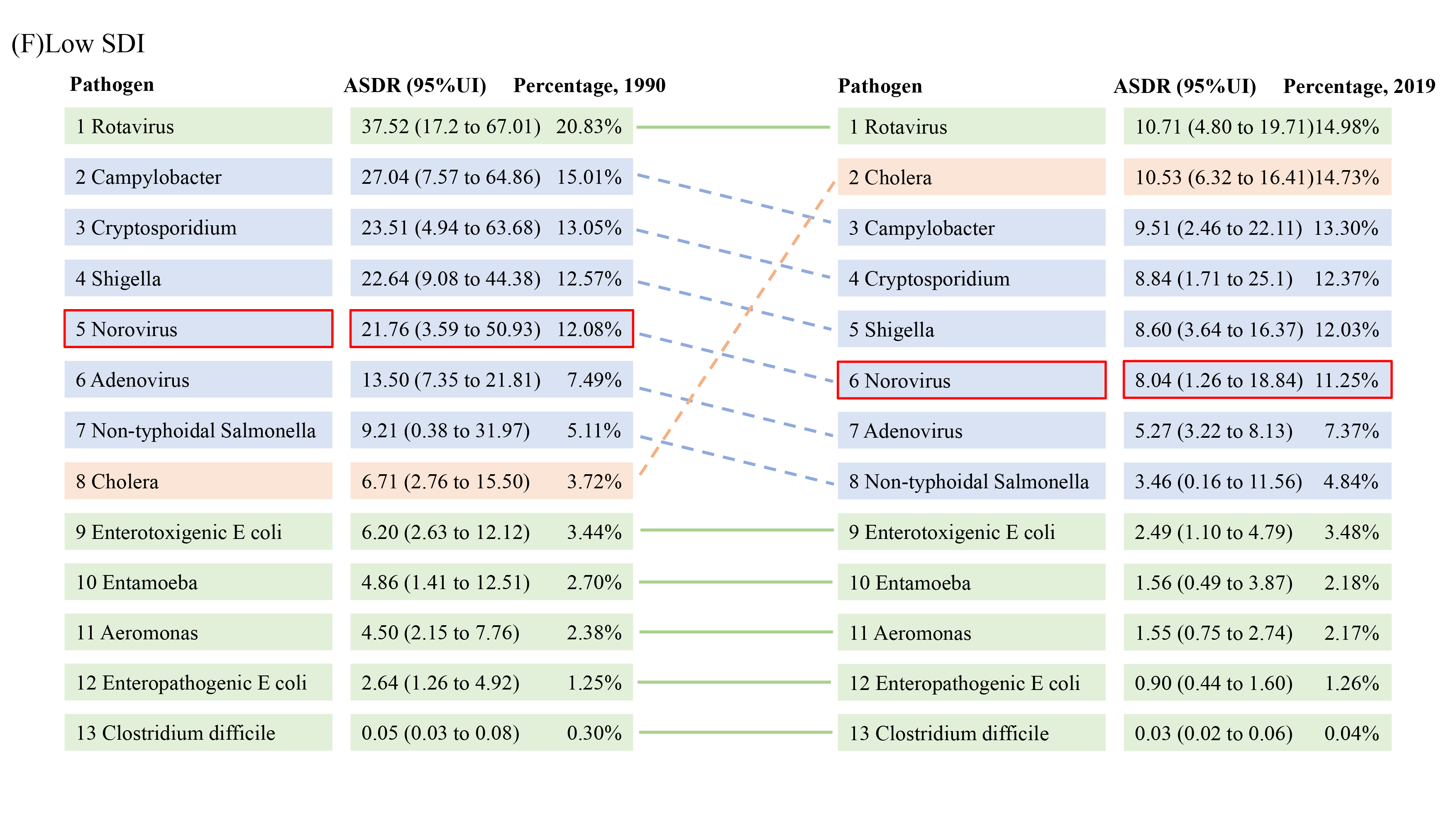

Supplement: Supplementary file 1 [file Data_Sheet_1.ZIP › Supplemental Materials/Supplementary Figure.S12.tif]

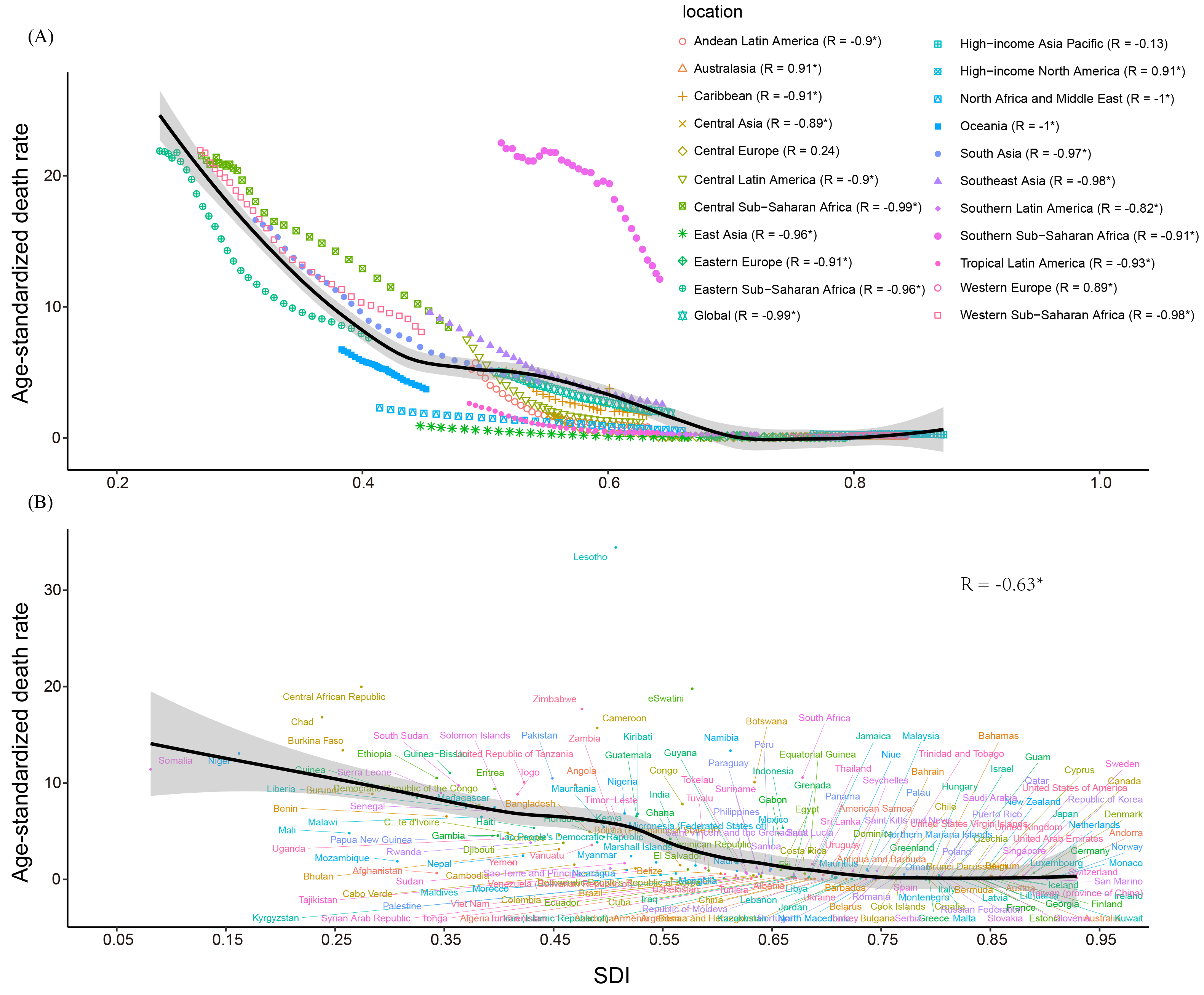

Supplement: Supplementary file 1 [file Data_Sheet_1.ZIP › Supplemental Materials/Supplementary Figure.S13.tif]

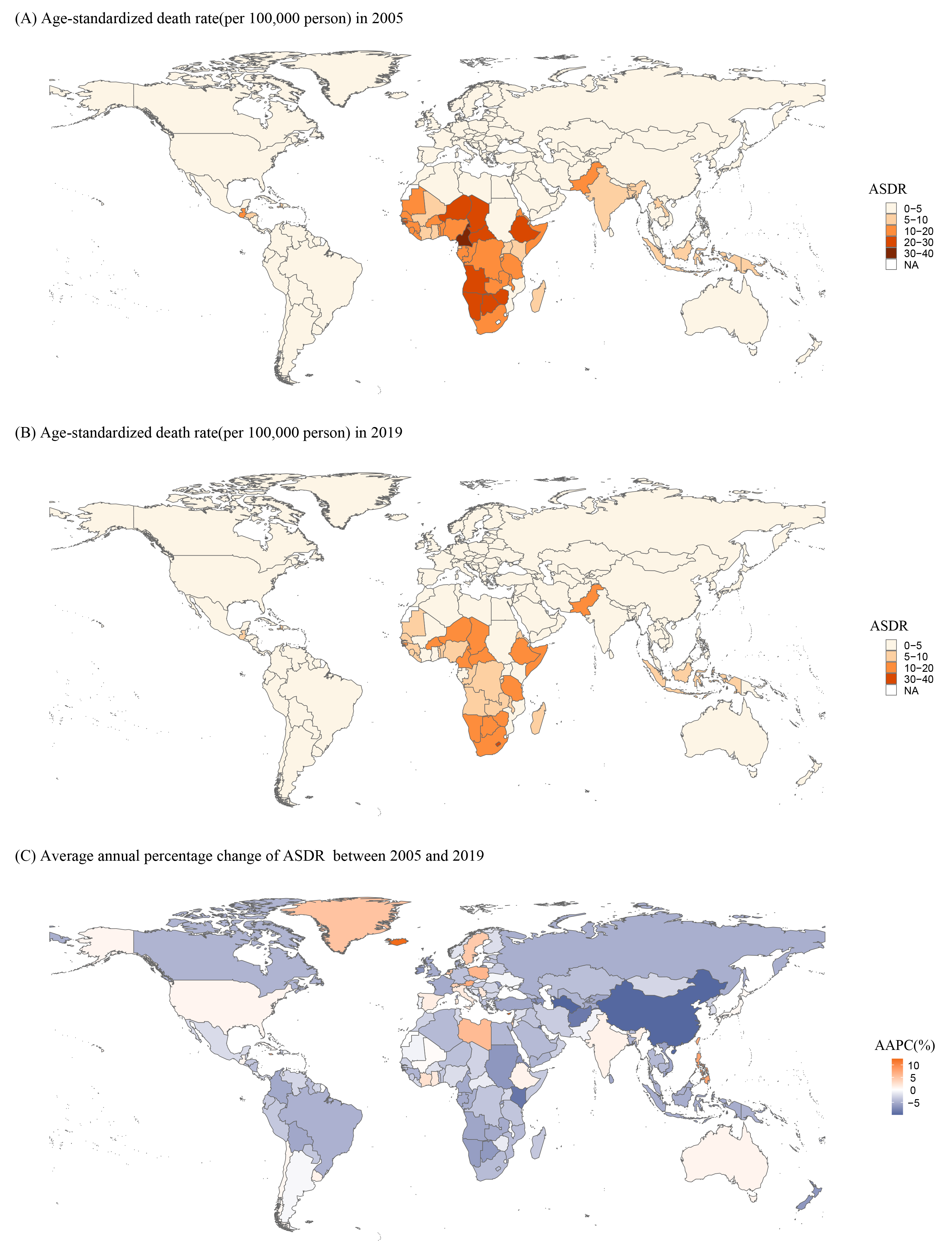

Supplement: Supplementary file 1 [file Data_Sheet_1.ZIP › Supplemental Materials/Supplementary Figure.S14.tif]

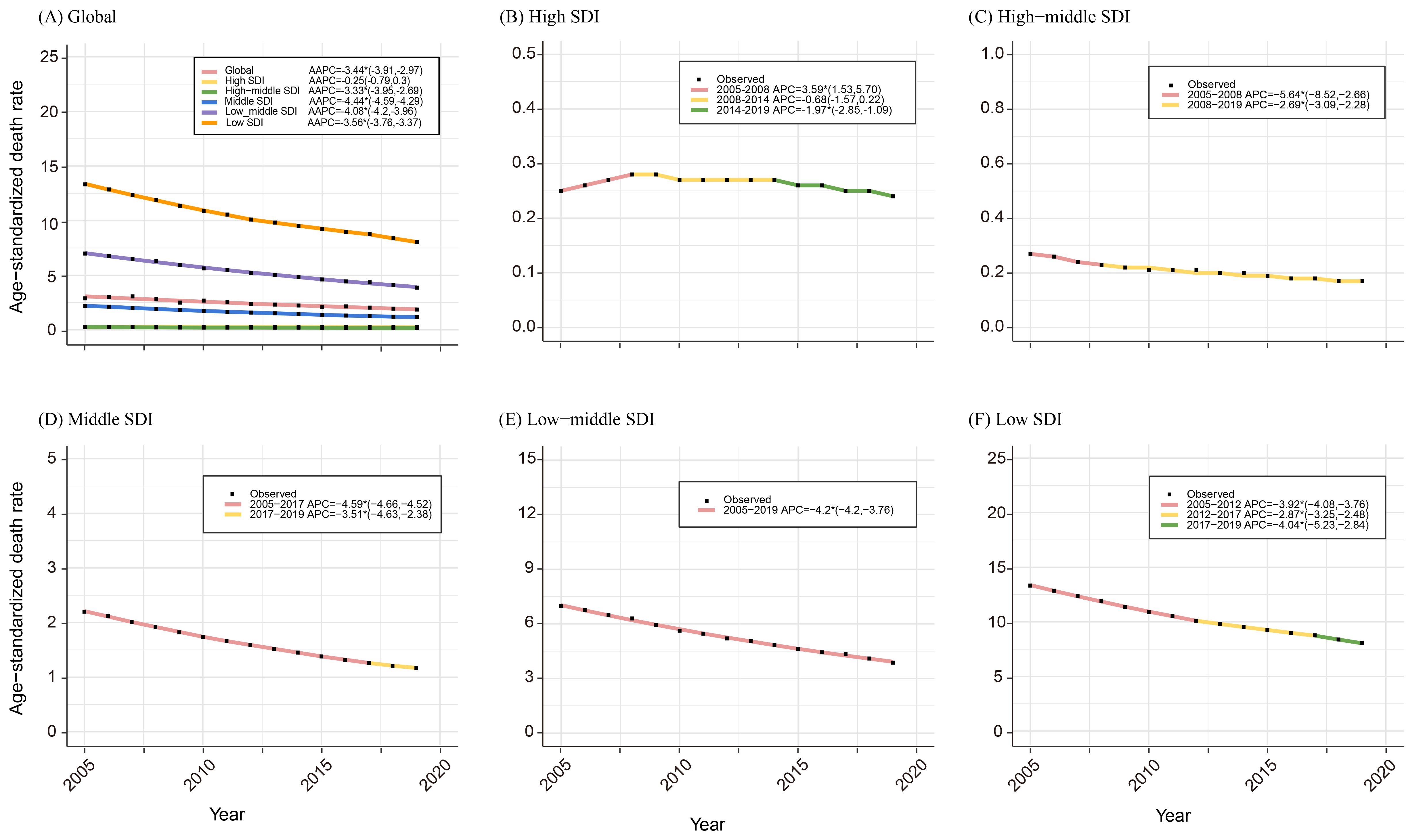

Supplement: Supplementary file 1 [file Data_Sheet_1.ZIP › Supplemental Materials/Supplementary Figure.S15.tif]

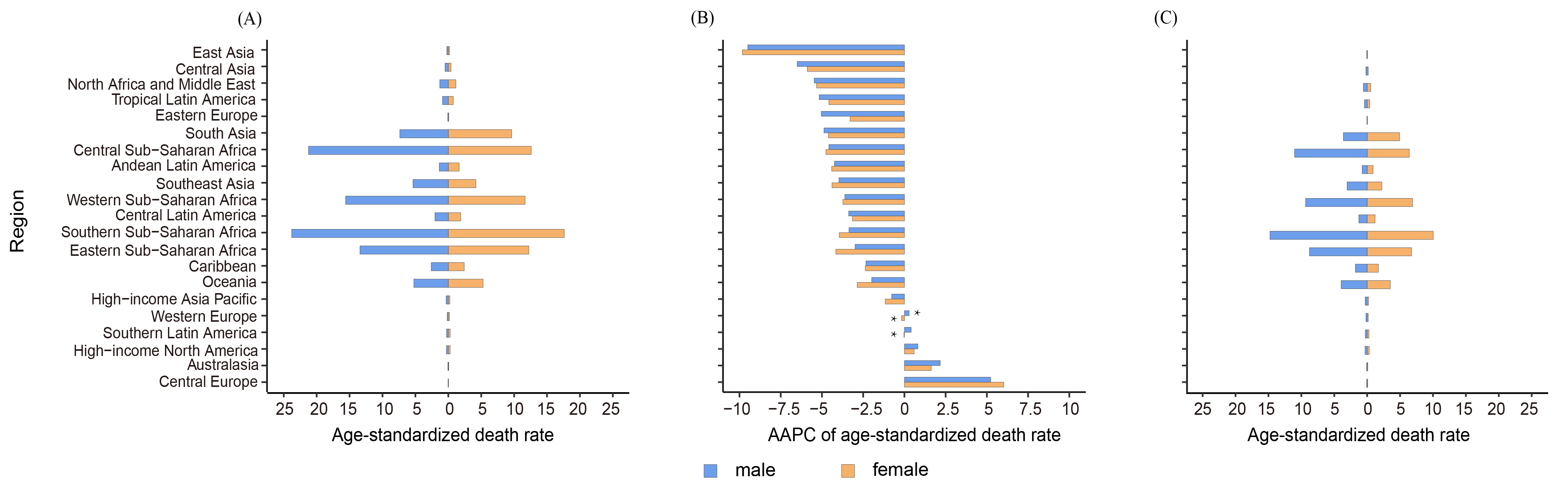

Supplement: Supplementary file 1 [file Data_Sheet_1.ZIP › Supplemental Materials/Supplementary Figure.S16.tif]

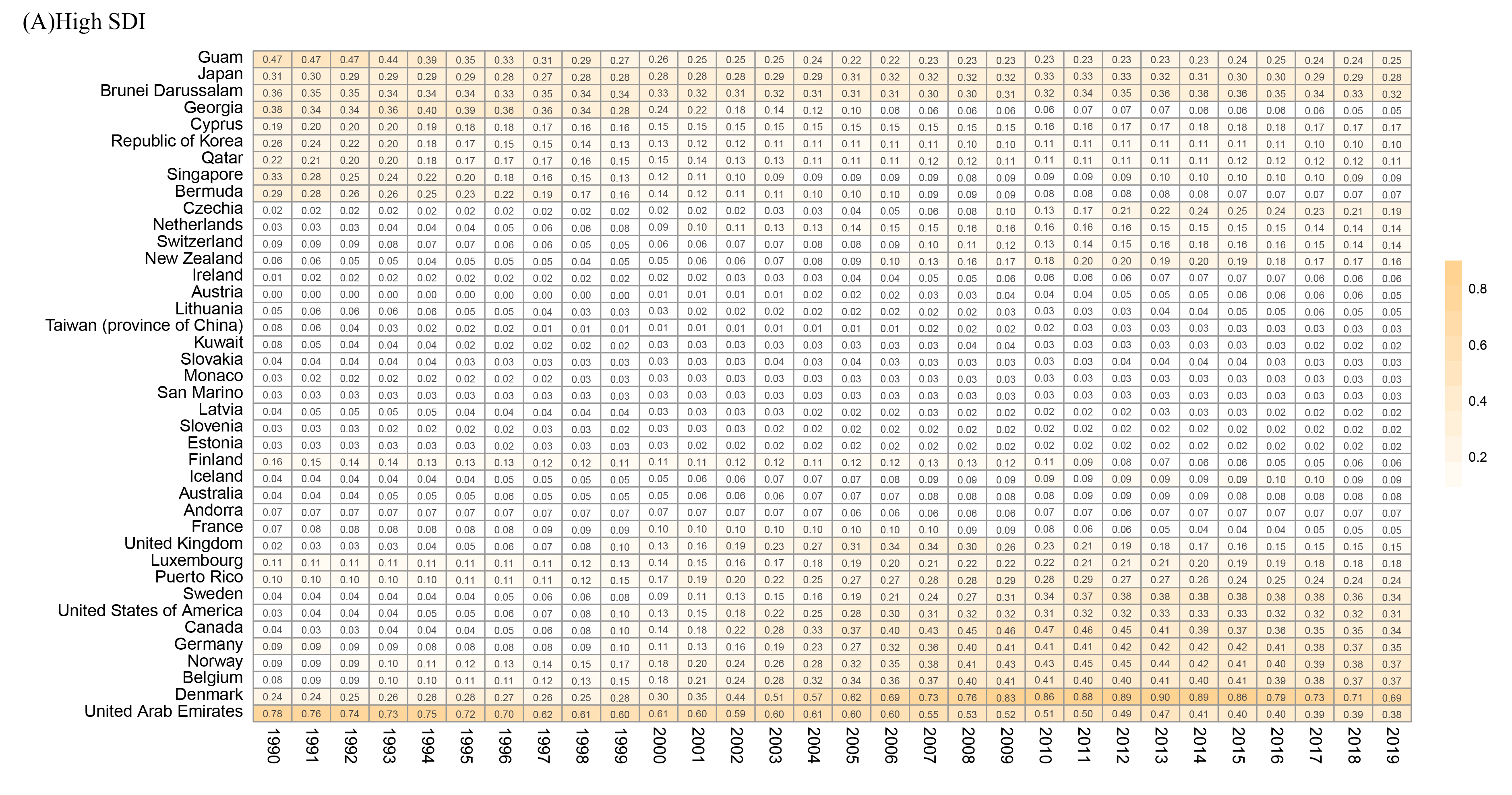

Supplement: Supplementary file 1 [file Data_Sheet_1.ZIP › Supplemental Materials/Supplementary Figure.S2.tif]

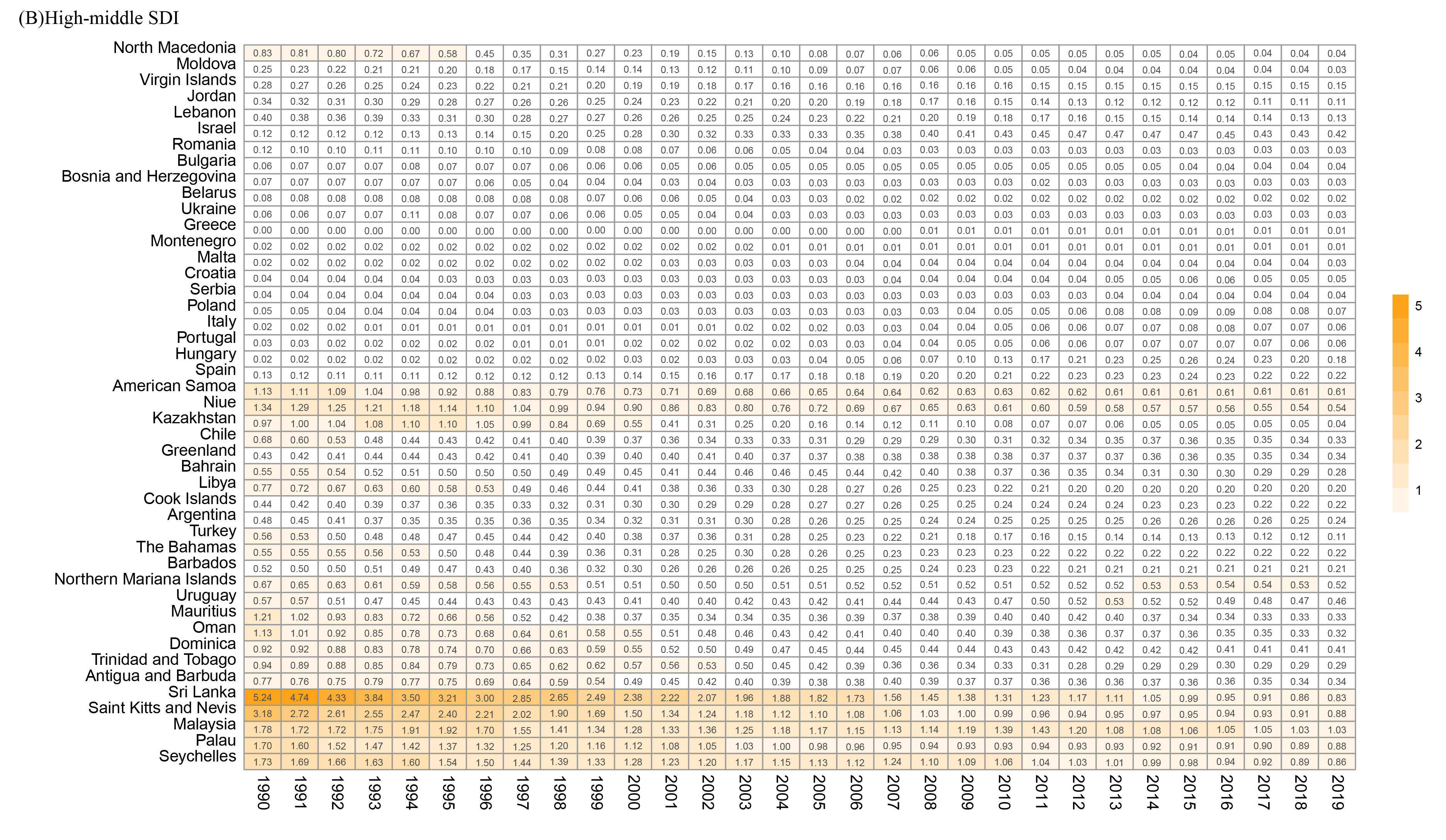

Supplement: Supplementary file 1 [file Data_Sheet_1.ZIP › Supplemental Materials/Supplementary Figure.S3.tif]

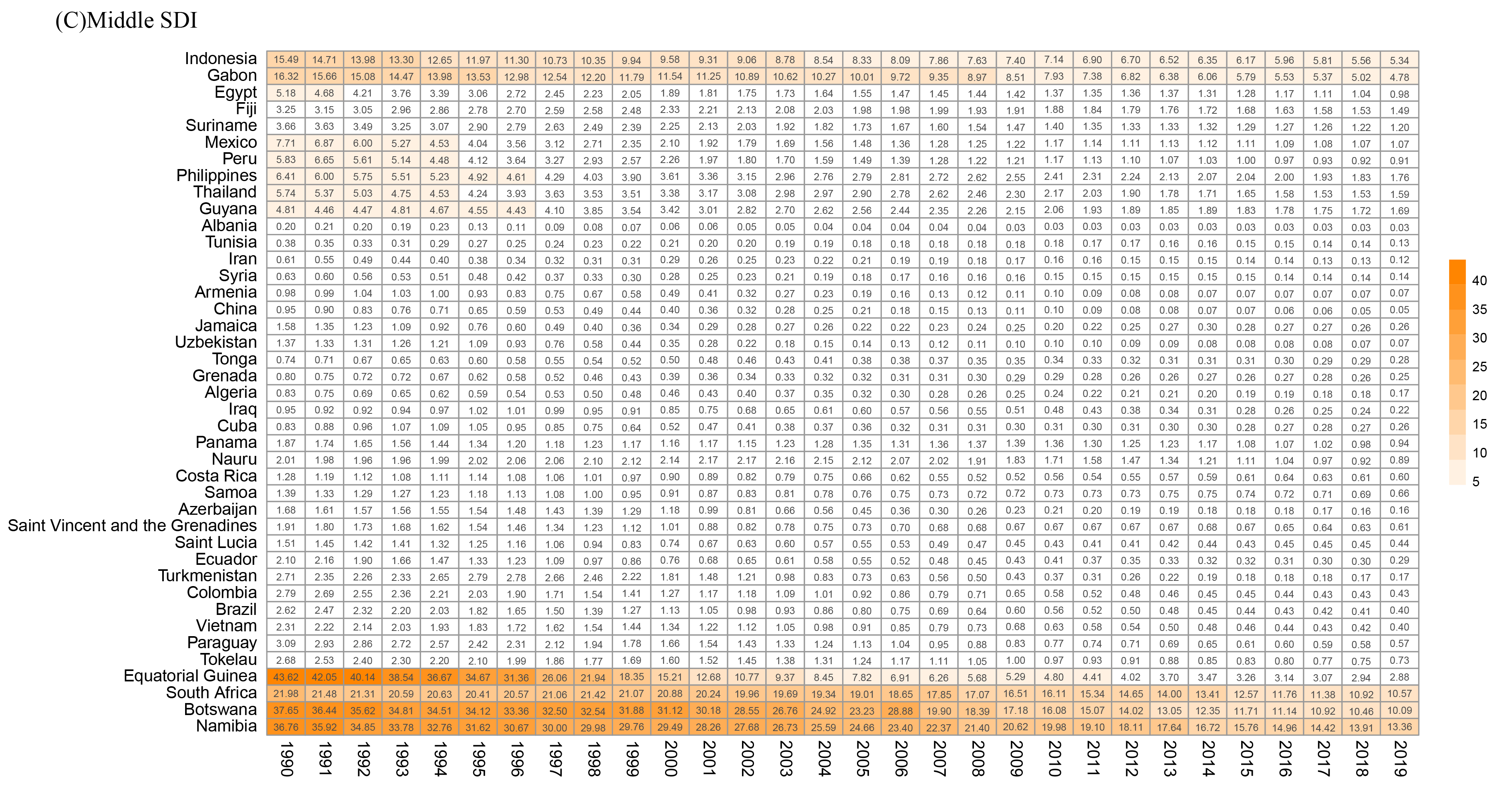

Supplement: Supplementary file 1 [file Data_Sheet_1.ZIP › Supplemental Materials/Supplementary Figure.S4.tif]

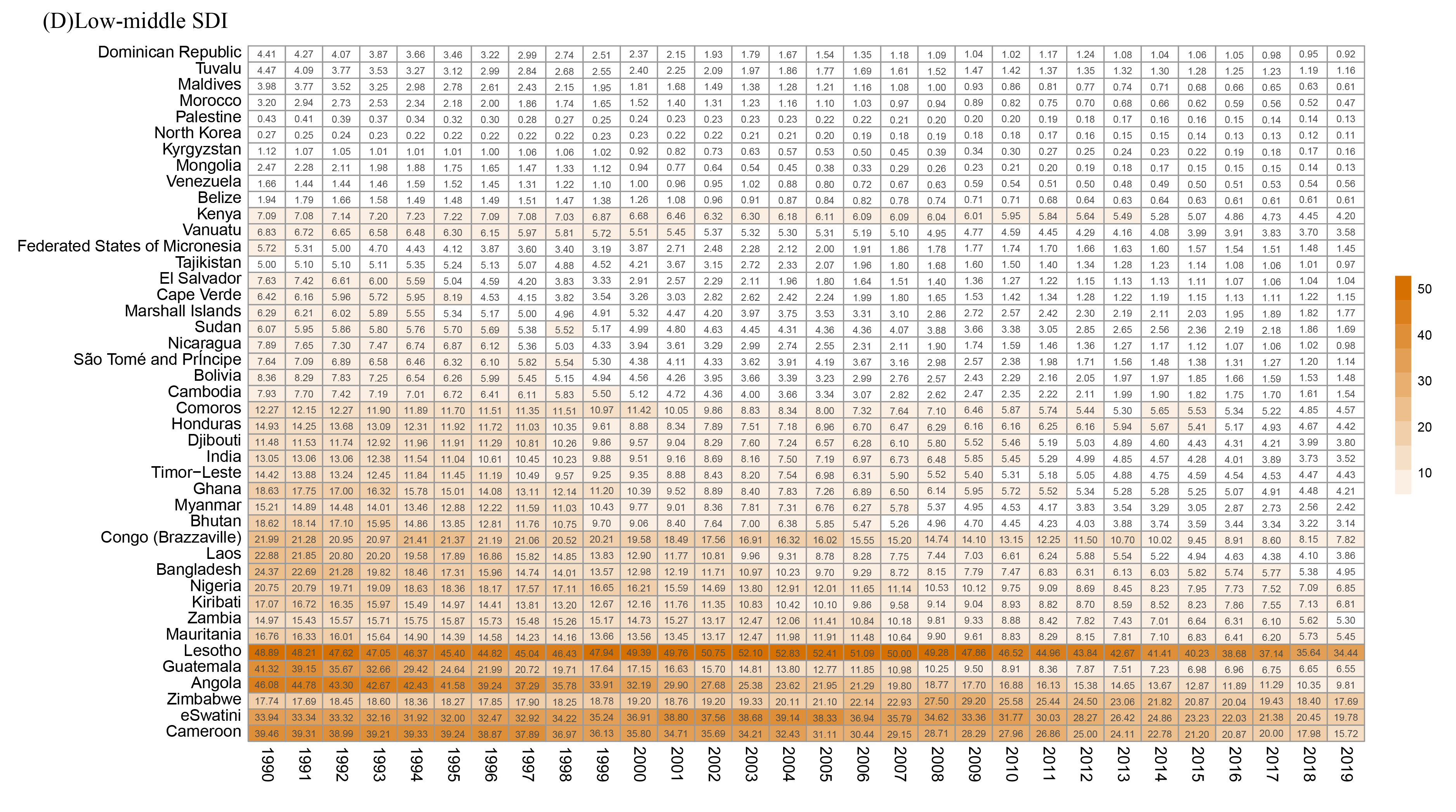

Supplement: Supplementary file 1 [file Data_Sheet_1.ZIP › Supplemental Materials/Supplementary Figure.S5.tif]

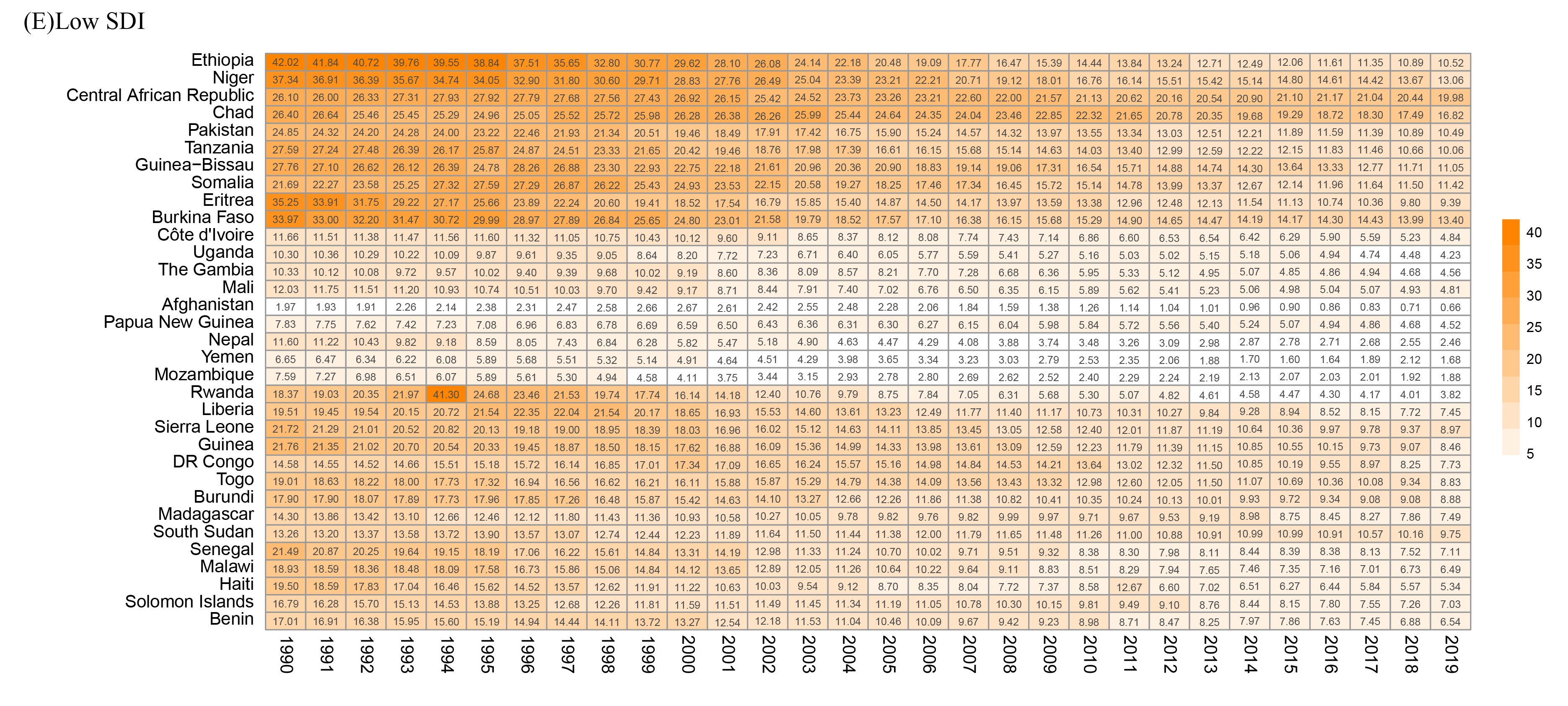

Supplement: Supplementary file 1 [file Data_Sheet_1.ZIP › Supplemental Materials/Supplementary Figure.S6.tif]

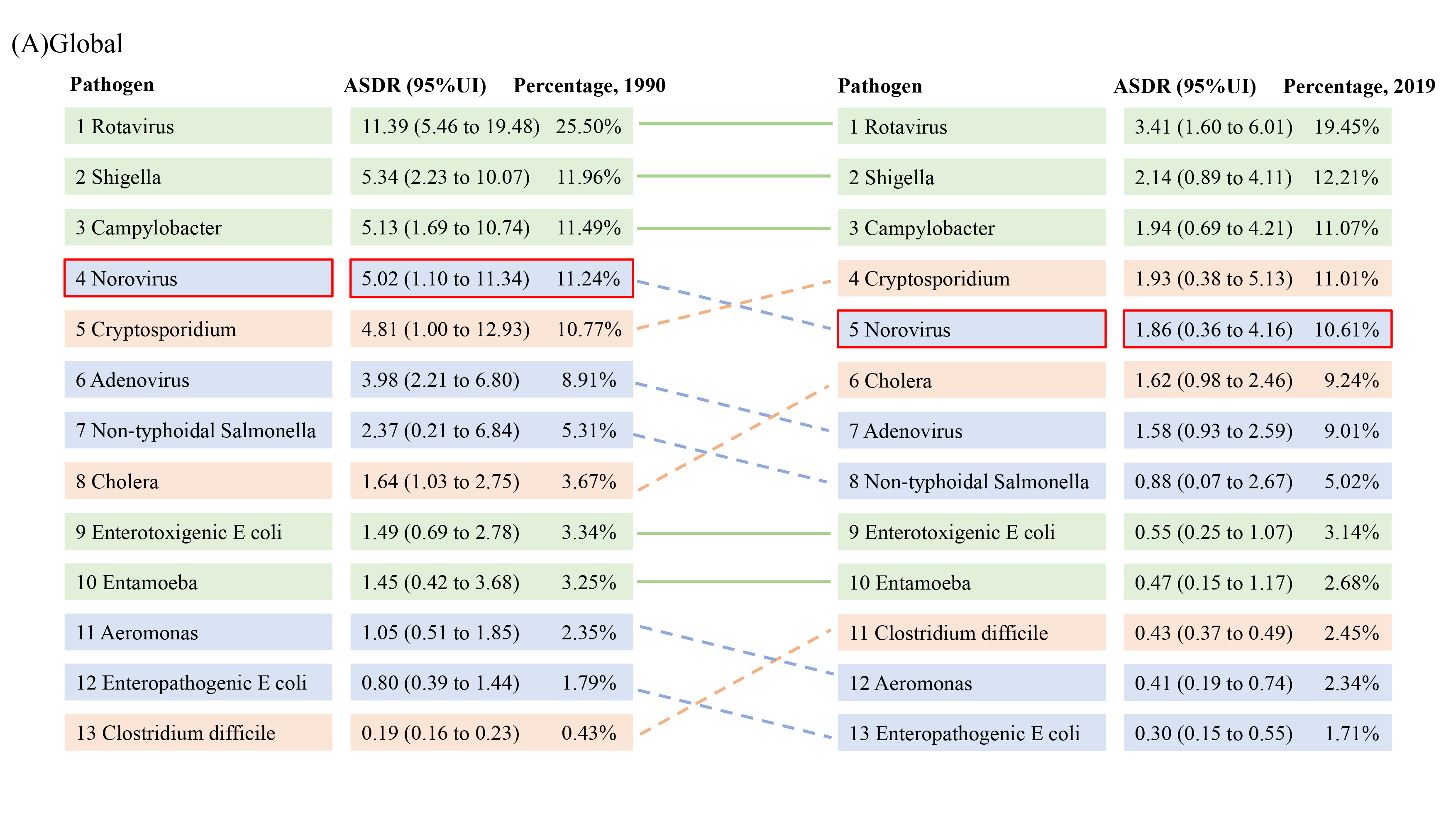

Supplement: Supplementary file 1 [file Data_Sheet_1.ZIP › Supplemental Materials/Supplementary Figure.S7.tif]

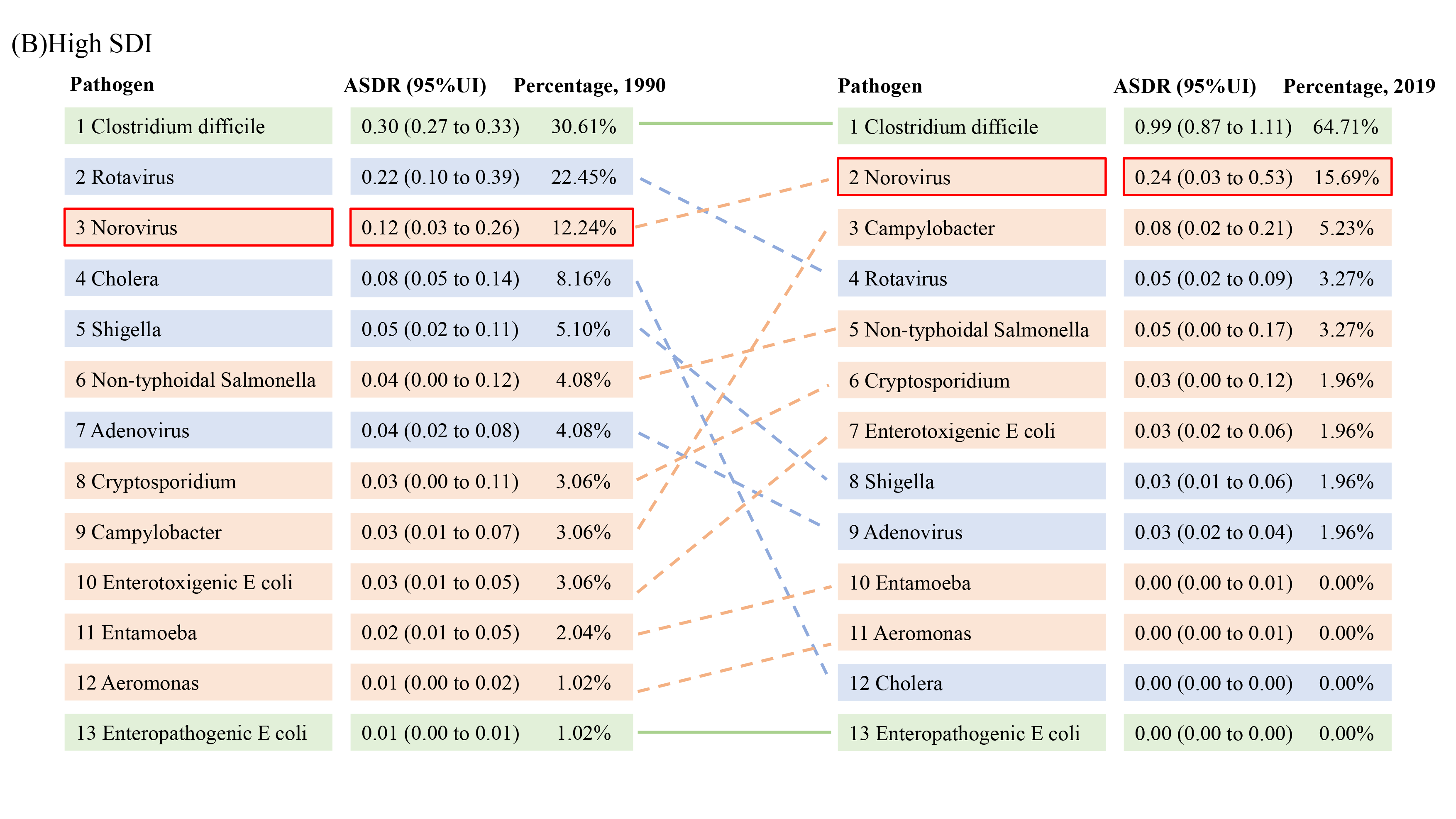

Supplement: Supplementary file 1 [file Data_Sheet_1.ZIP › Supplemental Materials/Supplementary Figure.S8.tif]

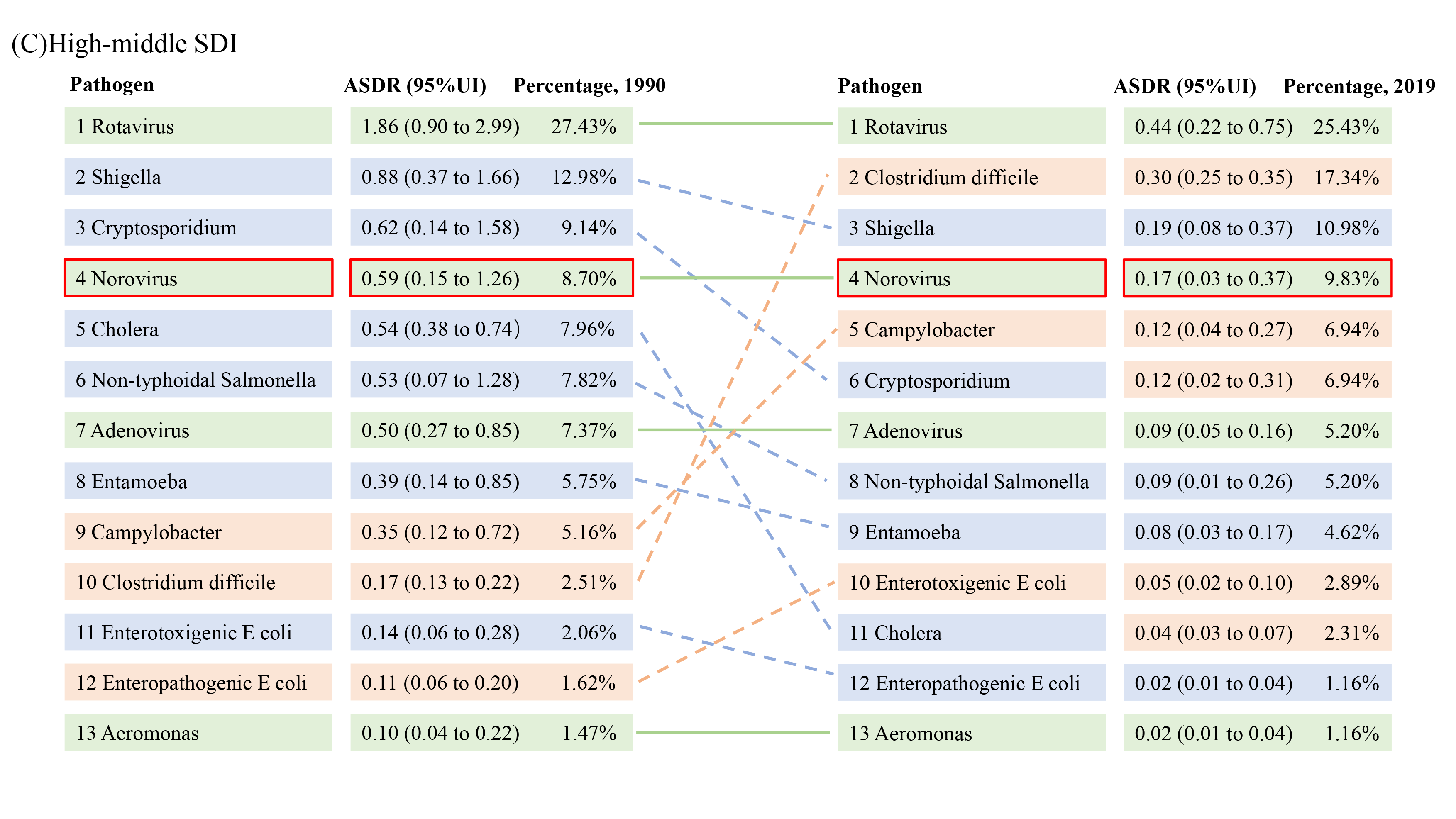

Supplement: Supplementary file 1 [file Data_Sheet_1.ZIP › Supplemental Materials/Supplementary Figure.S9.tif]
