# Supplementary material for: Global Burden and Trends of Norovirus-Associated Diseases From 1990 to 2019: An Observational Trend Study
Source: Front Public Health. 2022 Jun 17;10:905172. doi: 10.3389/fpubh.2022.905172 (PMC9247406; doi:10.3389/fpubh.2022.905172)
Supplement: Supplementary file 2 [file Data_Sheet_2.pdf]

Supplementary materials

Supplementary Figure S1. The ASDR of NADs in 21 GBD regions and the AAPC of ASDR between 1990 and 2019.

Supplementary Figure S2. The ASDR of NADs in high SDI regions over the 30 years.

Supplementary Figure S3. The ASDR of NADs in high-middle SDI regions over the 30 years.

Supplementary Figure S4. The ASDR of NADs in middle SDI regions over the 30 years.

Supplementary Figure S5. The ASDR of NADs in low-middle SDI regions over the 30 years.

Supplementary Figure S6. The ASDR of NADs in low SDI regions over the 30 years.

Supplementary Figure S7. The ASDR and percentages of 13 pathogens causing diarrheal diseases with percentage rankings in 1990 and 2019 globally.

Supplementary Figure S8. The ASDR and percentages of 13 pathogens causing diarrheal diseases with percentage rankings in 1990 and 2019 in high SDI regions.

Supplementary Figure S9. The ASDR and percentages of 13 pathogens causing diarrheal diseases with percentage rankings in 1990 and 2019 in high-middle SDI regions.

Supplementary Figure S10. The ASDR and percentages of 13 pathogens causing diarrheal diseases with percentage rankings in 1990 and 2019 in middle SDI regions.

Supplementary Figure S11. The ASDR and percentages of 13 pathogens causing diarrheal diseases with percentage rankings in 1990 and 2019 in low-middle SDI regions.

Supplementary Figure S12. The ASDR and percentages of 13 pathogens causing diarrheal diseases with percentage rankings in 1990 and 2019 in low SDI regions.

Supplementary Figure S13. Association between ASDR of NADs and SDI among 21 regions over 30 years and among 204 countries in 2019.

Supplementary Figure S14. Global burden of NADs in 2005 and 2019 with the annual percentage change rate over the 15 years. (A) ASDR in 2005; (B) ASDR in 2019; (C) AAPCs from 2005 to 2019.

Supplementary Figure S15. Temporal trends of ASDR globally and in different SDI regions over 15 years. (A) Global; (B) High SDI; (C) High-middle SDI; (D) Middle SDI; (E) Low-middle SDI; (F) Low SDI. The APCs with asterisks (*) are statistically significant ($P < 0.05$).

Supplementary Figure S16. The ASDR of NADs in 21 GBD regions and the AAPC of ASDR between 2005 and 2019. (A) ASDR in 2005; (B) AAPCs between 2005 and 2019; (C) ASDR in 2019. The AAPCs with asterisks (*) are not statistically significant ($P \geqslant 0.05$).
